# Unlocking the biomineralization style and affinity of Paleozoic fusulinid foraminifera

**DOI:** 10.1038/s41598-017-15666-1

**Published:** 2017-11-09

**Authors:** Zofia Dubicka, Przemysław Gorzelak

**Affiliations:** 1 University of Warsaw, Faculty of Geology, Żwirki i Wigury 93, 02-089 Warsaw, Poland; 2 University of Silesia, Faculty of Earth Sciences, Będzińska 60, 41-200 Sosnowiec, Poland; 3 Institute of Paleobiology, Polish Academy of Sciences, Twarda 51/55, 00-818 Warsaw, Poland

## Abstract

Fusulinids are the most diverse, abundant and geographically widespread Paleozoic foraminifera which are widely considered to possess a “homogeneously microgranular” test microstructure composed of subangular grains of several micrometers in size. However, this texture appears to be a diagenetic artifact. Here we describe well-preserved Devonian calcareous fusulinids (*Nanicella*) from the Holy Cross Mountains (HCM) in central Poland. Foraminifera from Poland in which the primary nature of tests have not been masked by diagenesis are composed of low magnesium calcite spherical grains up to about 100 nanometers in diameter, identical to those observed in Recent and fossil hyaline foraminifera (Rotaliida, Globothalamea). These data call the paradigm of microgranular test microstructure of Foraminifera into question, and suggest a possible phylogenetic relationship between globothalamids and some fusulinids.

## Introduction

Foraminifera are among the most important microorganisms in the earth sciences because they constitute a valuable tool for paleoenvironmental reconstructions and stratigraphic analyses. Traditionally, the higher-level taxonomy of Foraminifera is based on test structure^1–4^. The most common calcareous foraminiferal wall textures are hyaline (Rotaliida), porcelanous (Miliolida), and exclusively microgranular in Paleozoic forms (Fusulinata). The class Fusulinata (taxonomic rank after Vachard^5^ and Vachard *et al*.^6^), defined as a group possessing a “homogeneously microgranular” test composition^2–4,7,8^ consisting of closely packed subangular grains several micrometers in size^7,9,10^, is the most abundant group of Paleozoic foraminifera. It comprises several hundred genera^11,12^, including all Paleozoic calcareous taxa except representatives of Miliolida and Lagenida (Nodosariata)^6^. Although the term *microgranular* has been used for decades to characterize Paleozoic fusulinid test walls, the question of whether this texture is of primary or diagenetic origin has never been properly resolved. Surprisingly, their wall structure has been investigated mostly under low-resolution light microscopes (e.g. Rigaud *et al*.^13^); scanning electron microscopy (SEM) has been used only rarely^8–10^. Furthermore, most studies of Paleozoic foraminifera have been based on recrystallized specimens characterized by obscure original test compositions (see also Mikhalevich^14^). Such specimens typically display micrometer-sized neomorphic calcite/overgrowths on test surfaces and within their interiors^8–10,15^. Notably, observations of such micrometer-sized particles within microgranular textures have led to discussions as to whether they were secreted or agglutinated^7,10,13,16–18^.

There has been no presentation to date of micro- or nanoscale structural observations coupled with geochemical characteristics of Paleozoic foraminifera. To bridge this gap, in this paper we employed various analytical tools to characterize Paleozoic foraminiferal tests. Our material comprises exceptionally well-preserved Devonian calcareous nanicellid foraminifera from the Kowala section of the Holy Cross Mountains (HCM) in central Poland. Our findings provide new insights into the biomineralization style and affinities of Paleozoic “fusulinid” foraminifera, and invalidate the “microgranular fusulinids” paradigm.

## Results

**Figure 1:**
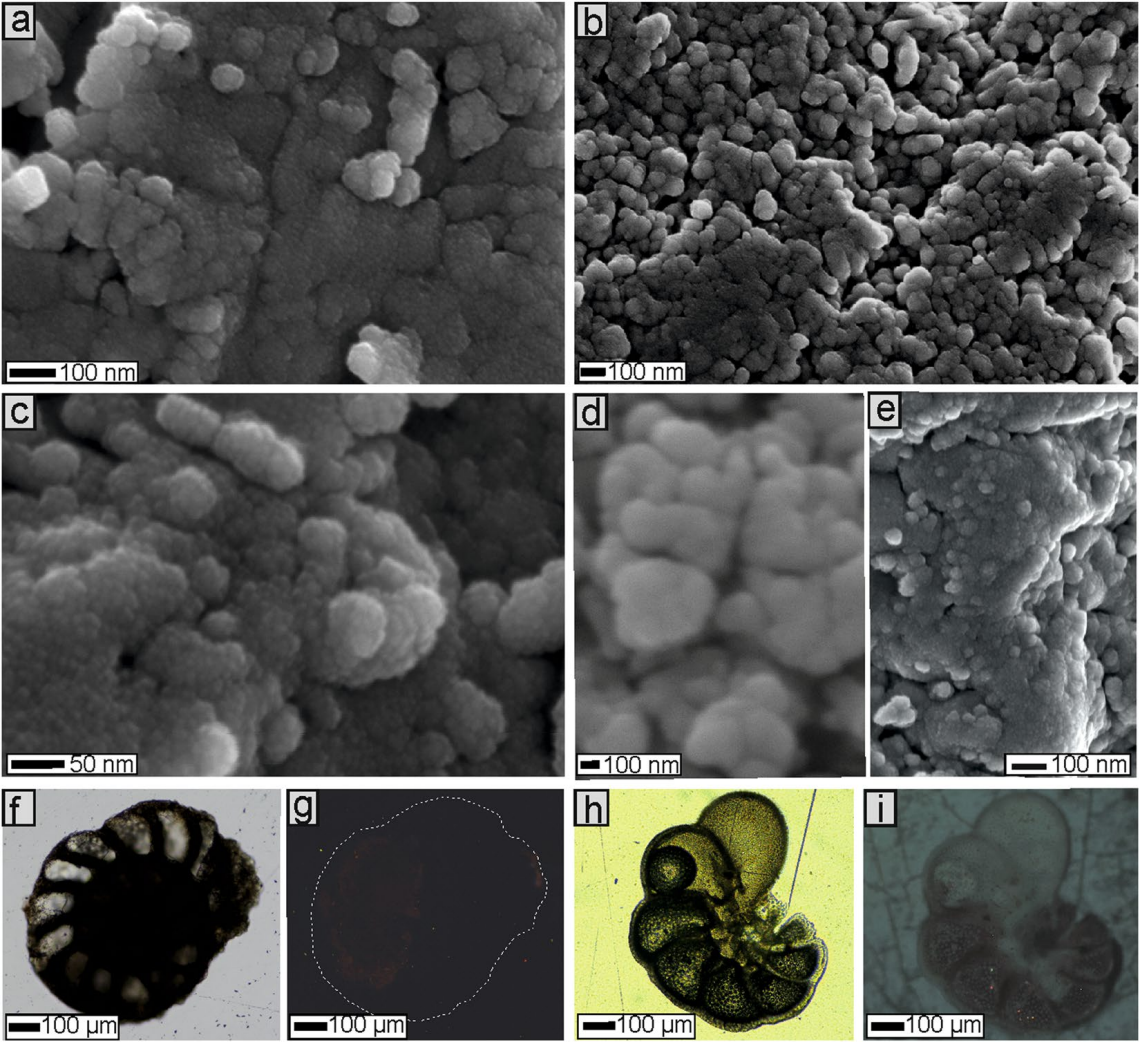
FESEM images (**a**–**e**) illustrating wall structure details in nanoscale of Recent *Cibicides* (**b**,**d**), Cretaceous *Pseudouvigerina* (**e**) and Devonian *Nanicella* (**a**,**c**). Optical microscopy (**f**,**h**) and cathodoluminescence images (**g**,**i**) of Recent *Cibicides* (**h**,**i**) and well-preserved Devonian *Nanicella* (**f**,**g**).

The specimens at hand appear not to have been significantly altered by diagenesis, as suggested by the absence of any neomorphic calcite crystal overgrowths (Fig. 1a,c), which are characteristic of diagenetically altered foraminiferal tests^15^. The absence of extensive diagenetic changes is supported by cathodoluminescence analyses. Recent low-Mg calcite foraminifers which grow in the water column (planktonic or epibenthic forms) do not reveal Mn^2+^-activated cathodoluminescence^19,20^ (Fig. 1h). The orange-red luminescence of hyaline foraminiferal tests can only be displayed by some infaunal benthic foraminifera due to the incorporation of Mn^2+^ in a specific microhabitat within the sediment. Studied epibenthic Devonian nanicellids, in turn, are either non-luminescent or show rare spots of very dull orange luminescence (Fig. 1f); accordingly, they can be classified as well preserved. However, caution has to be taken when using cathodoluminescence as a tool to interpret diagenetic changes, because Mn-activated CL may be quenched by Fe. Nevertheless, although Fe was recorded in foraminiferal tests by electron microprobe, its concentration is very low (0.02–0.03 wt%), below detection limit (>0.05 wt%) (Supplemental Table 1). Likewise, no detectable Mn or other diagenetic elements (e.g., Na) were noted.

Overall, the results of geochemical analyses show that Devonian foraminifers are preserved as low-magnesium calcite. Their mean Mg contents of 0.36 and 0.42 wt% correspond to a Mg/Ca ratio of 15‒17.69 (mmol/mol), and fall within the range of the magnesium content of Recent rotaliids (e.g., Bentov and Erez^21^; see Discussion below). Admittedly, the admixture of strontium is generally lower, i.e., 0.5‒0.6 wt%, corresponding to a Sr/Ca ratio of 0.57‒0.72 (mmol/mol), than in living representatives, which are typically characterized by Sr/Ca ratios of >1. Given that Sr/Ca ratios generally decrease with progressive diagenetic alteration (e.g., Edgar *et al*.^22^), the occurrence of fine-scale diagenetic changes in these foraminifera cannot be excluded. Nevertheless, the use of Sr/Ca ratio as a diagenetic marker can be problematic (e.g., Ullmann and Korte^23^). Notwithstanding, it has been argued that diagenetic depletion of trace elements in foraminiferal tests may occur without visible textural changes^24^. Indeed, our specimens maintain a primary nanocomposite structure, with spherical nanograins, up to about 100 nm in size, identical to those observed in Recent and Mesozoic hyaline foraminifera (Globothalamea)^25,26^.

## Discussion

The three main groups/classes of Recent calcareous foraminifera, distinguished based on molecular data^27^, i.e., Globothalamea, Tubothalamea, and Lagenida (the latter group probably constitutes a distinct class as well), display significantly different textural characteristics, following distinct calcification processes^28^. Of these groups, only the calcification mechanism in Lagenida remains unexplored. The wall microstructure of rotaliids (Globothalamea) (including buliminids and planktonic foraminifera), commonly referred to as hyaline, is composed of irregularly arranged spherical calcitic biocrystallites, the so-called “nanograins” up to 100 nanometers in diameter separated from each other by space of a few nanometers^25,26^ (Fig. 1b,d). These nanograins are generally grouped into irregular aggregates measuring up to several µm across. A rotaliid test is composed of low-magnesium calcite, for which Mg/Ca ratios are around 1–20 mmol/mol^29–32^. New calcite biocrystallites are formed extracellularly, with the involvement of an organic template consisting of proteins and polysaccharides, i.e., the so-called Organic Primary Envelope (OPE), Primary Organic Temple (POT), Primary Organic Membrane (POM) or Primary Organic Sheet (POS)^26,28,31,33–37^.

**Figure 2:**
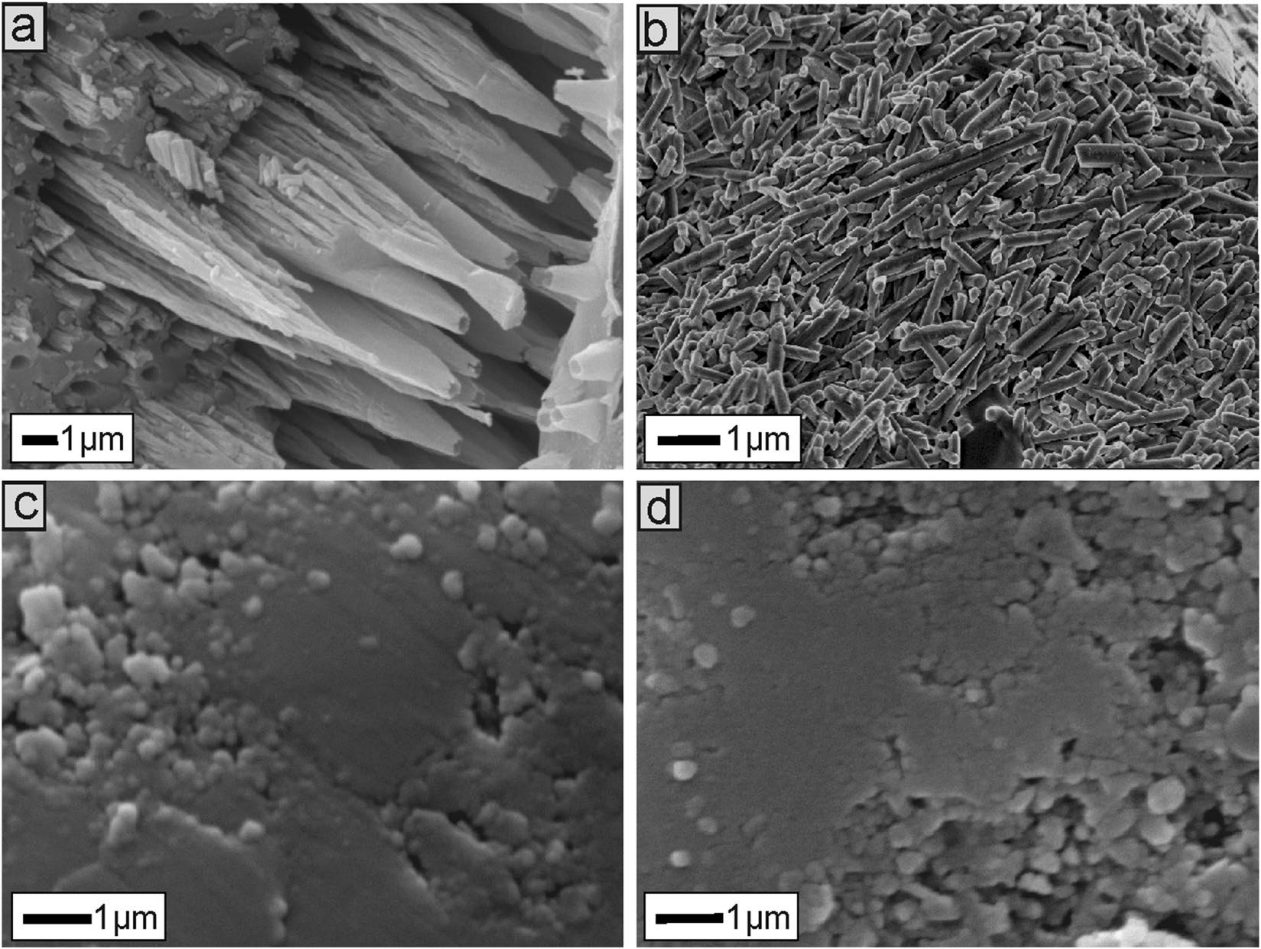
SEM images illustrating wall structure details in microscale of Recent *Nodosaria* (Lagenida) (**a**), *Triloculina* (Miliolida) (**b**) and *Cibicides* (Rotaliida) (**d**) as well as Devonian *Nanicella* (**c**).

In contrast, miliolid walls (Tubothalamea), known as porcelanous, are composed of thick layer of needle-shaped biocrystallites^26,38–40^ up to 1 μm in length and over 100 nm in width, arbitrarily arranged and separated from each other by a space ranging from several to 100 nm (Fig. 2b). Calcite biocrystallites in these foraminifera^25,26,31,34,37,41^ nucleate inside cytoplasmic vesicles that are transported outside the test and congregate in the chamber wall within an organic matrix. Miliolid biocrystallites are composed of high-magnesium calcite, for which Mg/Ca ratios are about 100–150 mmol/mol.

Lagenids (Lagenida) display biocrystalline test texture different from that of other calcifying foraminifera, composed of tightly-packed single*-*crystal bundles oriented perpendicularly to the test wall (Fig. 2a). Each calcite biocrystal possesses an inner pore, extended along the whole length of the biocrystal, which is probably related to cytoplasmic flow and test secretion processes. However, to date no precise calcification mechanisms of Lagenida have been identified.

The differences in calcification processes between porcelanous and perforate hyaline foraminifera are consistent with molecular data implying independent origins for Globothalamea (which includes hyaline rotaliids) and Tubothalamea^27,42^ (including porcelanous miliolids); their divergence probably dates as far back as the Neo-Proterozoic^43^. Because molecular studies of extinct forms are impossible, Fusulinida is classified as *incertae sedis* by Pawlowski *et al*.^27^; however, these authors pointed out that this order may be partly associated with Globothalamea and Tubothalamea. This assumption contradicts the traditional opinion that “foraminifera with microgranular walls became extinct at the end of Permian and left no descendants, thus both actualistic and taxonomic uniformitarian approaches to the study of fusulinid wall morphology and paleobiology fail” (Hageman and Kaesler^10^ p. 181).

Our fine-scale observations of nanicellid tests do not reveal any of the features of the “microgranular test”, and confirm, for the first time using SEM, previous hypothesis^44^ that the wall of these foraminifers is hyaline. Indeed our data show that nanicellid tests are entirely composed of spherical low-magnesium calcitic nanograins up to about 100 nanometers in diameter, which merge into micrometer-sized irregular aggregates. Accordingly, they display exactly the same test structure and mineralogy as Recent and fossil hyaline foraminifera (Globothalamea)^25,26^. Admittedly, however, this low Mg content may be due to other factors such as the low Mg/Ca seawater ratio of the Devonian “calcitic sea” (e.g., Stanley^45^) or fine-scale diagenetic depletion of Mg. Nevertheless, the potential nanicellid (Nanicellidae) phylogenetic connection with Globothalamea can be additionally supported by general test morphology (chamber morphology, mode of coiling, foraminal distance), which has recently been postulated as the primary feature in higher-level taxonomy^27^. Nanicellids, similar to other representatives of the class Globothalamea, possess multichambered, trochospirally enrolled tests with semi-globular chambers, and with a minimal foraminal distance between a given aperture and the last foramen^46^ (Fig. 1e). Indeed, the phylogenetic relationships of some fusulinids to Rotaliata^13,47,48^ and Textulariida^13,17,47^ (both taxa are within Globothlamea) have been previously emphasized. Nevertheless, to robustly test this hypothesis future phylogenetic analyses and in-depth structural studies on other well-preserved fusulinid taxa from various stratigraphic intervals (late Paleozoic, in particular) and post-Paleozic globothalamids are needed.

## Materials and Methods

Planispiral nanicellids are common in the Givetian to Frasnian bank-to-reef complex of the Holy Cross Mountains (HCM) in central Poland^49,50^. Isolated specimens of *Nanicella* were derived from samples collected from marly-shale intercalations of the stromatoporoid-coral limestones of the Kowala railroad cut section (set B *sensu* Szulczewski^51^; Kowala Formation; see Racki^52^). This locality is well-known because of exceptionally low thermally altered organic matter^53,54^. Foraminifera were extracted from the friable sediments by washing through a 60-μm sieve. The material is stored in the S. J. Thugutt Museum of Geology, University of Warsaw (MWGUW ZI/67/43).

Hand-picked isolated specimens, coated with carbon, were examined with a field emission scanning electron microscope (FESEM) at the Institute of High Pressure Physics (Unipress), Polish Academy of Sciences in Warsaw.

The chemical composition of three selected nanicellid specimens was investigated using a CAMECA SX 100 electron microprobe (EMP) on uncovered, polished, carbon-coated thin sections at the Polish Geological Institute ‒ National Research Institute in Warsaw, Poland. The following conditions were applied: beam diameter: ~5 μm; accelerating voltage: 15 kV; beam current: 5 nA for calcium, and 20 nA for other elements; number of spot analyses: at least 3 per specimen. Thin sections were also examined using cold-cathode cathodoluminescence microscopy (an optical microscope coupled with a Cambridge Image Technology Ltd. CCL Mk5-2) in the Electron Microprobe Laboratory at the Polish Geological Institute – National Research Institute.

## Electronic supplementary material

Supplementary information 
