# Supplementary material for: Unlocking the biomineralization style and affinity of Paleozoic fusulinid foraminifera
Source: Sci Rep. 2017 Nov 9;7:15218. doi: 10.1038/s41598-017-15666-1 (PMC5680253; doi:10.1038/s41598-017-15666-1)
Supplement: Supplementary file 1 — Supplementary information [file 41598_2017_15666_MOESM1_ESM.doc]

**Unlocking the biomineralization style and affinity of Paleozoic fusulinid foraminifera**

**Zofia Dubicka**1,***, and Przemysław Gorzelak**2

1University of Warsaw, Faculty of Geology, Żwirki i Wigury 93, 02-089 Warsaw, Poland

2Institute of Paleobiology, Polish Academy of Sciences, Twarda 51/55, 00-818 Warsaw, Poland

*corresponding author z.dubicka@uw.edu.pl


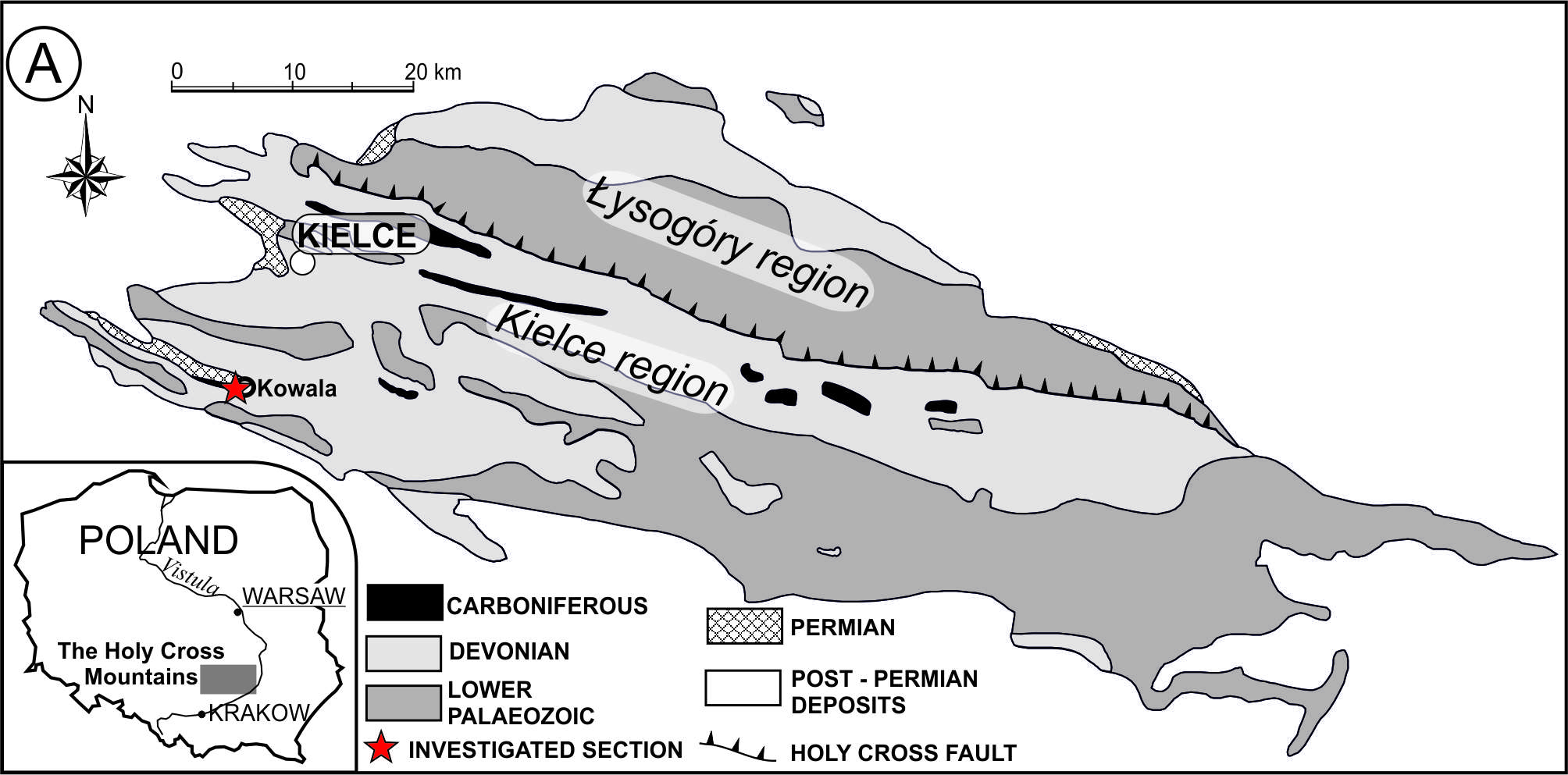


Figure S1. Location map redrawn from Marynowski *et al*.^54^ and originated through CorelDRAW Graphics Suite X7, [www.coreldraw.com](http://www.coreldraw.com/)

|  |  |  |  |  |  |  |  |
| --- | --- | --- | --- | --- | --- | --- | --- |
|  |  |  |  |  |  |  |  |
|  |  | specimen 1 | | specimen 2 | | specimen 3 | |
|  |  | range | mean | range | mean | range | mean |
|  | Ca | 39,2-39,3 | 39,25 | 39,02-39,32 | 39,23 | 39,01-39,35 | 39,24 |
|  | Fe | 0,01-0,05* | 0,03* | 0,01-0,03* | 0,02* | 0,01-0,05* | 0,03* |
|  | Mn | n.d. | - | 0-0,01* | - | 0-0,01* | - |
|  | Na | n.d. | - | 0-0,02* | 0,01* | 0-0,02* | 0,01* |
|  | F | n.d. | - | n.d. | - | n.d. | - |
|  | Mg | 0,32-0,41 | 0,36 | 0,36-0,48 | 0,41 | 0,36-0,43 | 0,39 |
|  | Sr | 0,04-0,07 | 0,05 | 0,05-0,07 | 0,06 | 0,04-0,06 | 0,05 |
|  | Si | 0-0,01* | - | 0-0,03* | 0,01* | 0,01-0,02* | 0,01* |
|  | Al | 0-0,02* | 0,01* | 0-0,02* | 0,01* | 0,01-0,02* | 0,02* |
|  | K | n.d. | - | n.d. | - | n.d. | - |
|  | Cl | 0-0,01* | 0,01* | 0-0,02* | 0,01* | 0-0,1* | 0,01* |
|  | S | 0,02-0,02 | 0,02 | 0,02-0,03 | 0,02 | 0,02-0,03 | 0,02 |
|  | P | n.d. | - | 0-0,01* | - | n.d. | - |
|  | Ba | 0-0,02 | 0,01 | 0-0,04 | 0,01 | 0-0,07 | 0,04 |
|  | O | 48,22-48,3 | 48,25 | 48,13-48,33 | 48,2 | 48,08-48,36 | 48,19 |
|  | *=detected but below detection limit; n.d.= not detected. | | | | |  |  |

Table S1. Results of geochemical analyses of Devonian foraminifers from Kowala (Poland).

|  |  |  |  |  |  |  |  |
| --- | --- | --- | --- | --- | --- | --- | --- |
